# The Agential View of Misfortune

**DOI:** 10.1007/s12110-024-09470-w

**Published:** 2024-03-20

**Authors:** Ronald J. Planer, Kim Sterelny

**Affiliations:** 1 School of Liberal Arts, University of Wollongong, Wollongong, Australia; 2 DFG Words, Bones, Genes, Tools Center, University of Tübingen, Tübingen, Germany; 3 Research School of Social Sciences, Australian National University, Canberra, Australia

**Keywords:** Sorcery, Witchcraft, Magic, Shamanism, Hyper-vigilance, Cultural evolution

## Abstract

In many traditional, small-scale societies, death and other misfortunes are commonly explained as a result of others’ malign occult agency. Here, we call this family of epistemic tendencies “the agential view of misfortune.” After reviewing several ethnographic case studies that illustrate this view, we argue that its origins and stability are puzzling from an evolutionary perspective. Not only is the agential view of misfortune false; it imposes costs on individuals and social groups that seem to far outweigh whatever benefits the view might provide. We thus doubt that the agential view of misfortune is explainable in terms of adaptive effects. However, neither does it seem readily explainable as a consequence of belief formation strategies that are on the whole adaptive (as is plausibly the case for certain other of our false beliefs, including some that are costly). Accordingly, we contend that the commonness of the agential view of misfortune demands a special evolutionary explanation of some kind. We provide a partial explanation of this phenomenon by highlighting the adaptive benefits that often flow to occult specialists in environments where the agential view of misfortune is entrenched. What this does not explain, however, is the general lack of resistance we observe in response to occultists’ exploitative behaviours over (cultural) evolutionary timescales. We conclude by canvassing a few possible explanations for this puzzling lack of resistance, and while we commit ourselves to none, we do find one option more promising than the others.

This article has two aims. The first is to make salient a widely documented phenomenon, but one which we believe is more puzzling than has been realised. This is belief in what we shall call “the agential view of misfortune.” In many pre-state, small-scale societies, a commonly cited explanation for death is other humans’ mystical or occult behaviour, even when that death is quite obviously the result of accident (e.g., a fall) or disease. Moreover, similar explanations are routinely offered for various non-fatal forms of misfortune, such as repeated hunting failure or the failure of a crop. For those who hold this view, then, serious—or in some instances, not so serious—instances of misfortune often prompt the suspicion that occult activity of some kind on the part of other humans is or may be to blame. People recognise different forms of such activity, and anthropologists have coined a variety of different terms to capture these distinctions, such as “sorcery,” “witchcraft,” and “evil eye.” Here, we shall frequently use the terms “malign magic” and sometimes “sorcery” as catchalls for all such forms of magic that are conceived of as harmful.

In many respects, the agential view of misfortune resembles other forms of superstitious thinking. In particular, it is shaped by the same principles of “sympathetic magic” (Frazer, 1890/1990) that shape many other forms of superstitious belief. One of these is the principle of contagion—the idea that certain objects or substances that were once in direct physical contact with a person bear a lasting connection to them which can be magically manipulated to affect that person. This principle forms the basis of the many forms of “leavings sorcery” which are common throughout Papua New Guinea, among many other places (see, e.g., Schwoerer, 2017, and the references therein). For example, men may worry that female sexual partners will conceal their semen and deliver it to a sorcerer (Read, 1954). Frazer’s other principle of sympathetic magic—the principle of similarity—holds that a resemblance between one object and another can form the basis of a magical relationship between the two. The most straightforward examples of malign magic based on this form of thinking are ones that involve the use of figurines or drawings of targets. Forms of “image sorcery” known from the Kimberleys and parts of Arnhem Land in Australia are clear examples (Berndt & Berndt, 1988). That the agential view of misfortune shares these principles in common with other forms of superstitious belief is no doubt of theoretical importance. However, it is also important that superstitious thinking can be common or even pervasive in the absence of the agential view of misfortune; they are not a package deal. For example, a 2017 survey of UK adults found that as much as 70% of respondents were unwilling to risk walking under a ladder, while 55% more generally described themselves as being superstitious (Vyse, 2019). We would imagine that few of these same respondents would be inclined to blame a family member or friend’s untimely death on others’ occult behaviour. Thus, the agential view of misfortune is not adequately handled simply by an explanation of superstitious belief in general.

Importantly: the agential view of misfortune is widespread geographically, ecologically, and economically. It is not confined to desert people, or those living in rainforest or high latitudes; it is found in foraging, farming, and herding communities. Singh (2021), following Hutton (2004, 2017), has shown that despite great variation in detail (albeit with some internal patterns), this general phenomenon is very widespread, if not quite universal, in small-scale societies, though perhaps one that is intensified by colonialism or other social crises.^1^ This tendency to explain misfortune in terms of other humans’ malign magic typically exists alongside other supernatural-explanatory tendencies;^2^ for example, misfortune may be mystically explained as a result of the violation of some group taboo or moral norm (so-called mystical retribution), or as the result of actions on the part of an occult being of some kind, such as a deity or deceased person (so-called spirit aggression or spirit violence), or some combination thereof.^3^ Indeed, according to Murdock (1980), spirit aggression is “the most common and widespread of all types of supernatural causation” (1980:20). In some cases these explanations compete. Denizens of small-scale societies are most reluctant to take a “shit happens” attitude towards illness and other misfortunes;^4^ occult forces of one kind or another *cause* shit to happen. These phenomena call out for special explanation, for the views they reflect are not just false, but expensive.^5^ Occult countermeasures are never free. We think this is especially true in the case of the agential view of misfortune—our focus here—with its implication of living persons as agents of harm. As will become clear below, such explanations directly create social tensions or outright conflict in ways that other explanations do not. Singh (2021) has pointed out these costs, though in our view without fully realising their consequences or the challenges they pose for theories of human social and cognitive evolution. The most important conclusion of this article is that the agential view of misfortune is inconsistent with even quite modest views of the power of selection to adaptively shape belief. By “selection,” we here have in mind natural selection operating on individuals’ genes and/or their cultural phenotypes. 6 In the case of the former, it is belief-formation mechanisms or strategies that are the natural candidates to undergo selection; in the case of the latter, selection might in addition operate on particular beliefs. In general, these modes of selectionist thinking provide a good account of belief formation processes. That is no surprise: what we think about the world matters for what we do in the world. It is against this backdrop that the agential view of misfortune stands out. This is true, as we show below, despite the fact that selection operates under constraint, and humans are not and could not be ideally rational belief-forming agents.

Our second aim is to offer a partial explanation for the agential view of misfortune: partial, because one crucial element in the dynamic we suggest is left unexplained. We will argue that despite the costs to communities and to most individuals in them, a salient minority benefits from this perspective on the world. Specialists in esoteric knowledge benefit from this belief system and the practices associated with it, and they have an interest in promoting it. However, although we sketch some options, we still lack an explanation for the apparent absence of “sales resistance” to the occultists’ pitch.

We structure the article as follows. The next section develops a richer picture of the target phenomenon, and of the puzzle this phenomenon presents, anchored around three ethnographic classics. “The Lessons of Ethnography” analyses those examples and argues that, collectively, they show that the agential view of misfortune requires special evolutionary explanation. “Side Effects” discusses one explanatory strategy: while the agential view of misfortune is costly, it is an inevitable side effect of cognitive heuristics that are overall adaptive (so the strategy contends); heuristics that have net benefits, despite some costs. We will claim that this strategy is unpersuasive and explain our scepticism. “Malign Magic” considers the idea that, despite its apparent costs, on balance the agential view of misfortune is beneficial. The line of thought here is similar to adaptive models of religious practice; though these religious practices impose some costs, those costs are outweighed by the benefits. The simple version of this idea fails, but we do extract from it the idea that for a salient minority the benefits do outweigh the costs. We conclude with the unsolved problem: how does this minority come to dominate the ideological landscape of the community of which they are a part? We suggest, but only in a preliminary way, three possible answers to this puzzle in “Sales Resistance” and then briefly conclude.

## Three Ethnographic Examples

Evans-Pritchard (1937) details the occult practices of the Azande, an African village culture with a mixed economy of agriculture and husbandry with some hunting. For the Azande, witchcraft is the go-to explanation for misfortune. According to Evans-Prichard, the Azande understand witchcraft as a “psychic emanation from witchcraft substance which is believed to cause injury to health and property” (1937:9). Witchcraft substance, in turn, is understood as “a material. . in the bodies of certain persons. It is discovered by autopsy in the dead and is supposed to be diagnosed by oracles in the living” (1937:9). Witchcraft is not learned, but inherited. Unlike sorcery, it does not depend on the use of “bad medicines” or “spells.”

For the most part, the Azande believe witchcraft to emanate from individuals within their own social network, with occult attack motivated by envy and allied emotions. The Azande have elaborate protocols to detect the source of actual and potential occult threat. The most reliable—the gold standard for revealing the source of occult danger—is the chicken oracle. The chicken oracle is consulted by asking a sacrificial chicken (or series of chickens) yes–no questions about danger and its sources. A consultant might want to know whether a prospective plan (marriage, travel, important economic decisions) would be at risk from occult threat, or who is responsible for some actual misfortune (a sick goat, hunting failure). A specialist makes and gives the chicken a measured dose of poison, and the chicken answers by living or dying. There are other oracles; for example, the termite oracle is consulted by inserting a twig into a termite nest, and the termites answer by eating or ignoring the offering. But these lesser probes are recognised to be fallible. One central theme of Evans-Prichard’s (1937) work is the pervasiveness of these practices. The threat of occult attack, and measures to mitigate the danger and reveal its source, are routine, regular features of life.

Knauft (2015) describes a very different culture, the Gebusi. Horticulturalist-foragers of lowland Papua New Guinea (PNG), the Gebusi are a rainforest people, and when Knauft did his original fieldwork in the 1970s, they were under very loose state control. There was some police presence in the region, but they were rarely or never seen in most villages. As with the other examples, the Gebusi believed that illness or accident were the result of occult attack. These dangers sometimes originated from strangers, outside their regular social circle, but they could well be from within that circle, the result of old grudges. As with the Azande, the Gebusi had clear protocols for identifying the source of attack. Sometimes (as the Gebusi saw things) the victims themselves would identify the guilty party. For the Gebusi mortuary rituals could require close interaction with the body. Since this was in the tropics, and the body was not kept cold, various exudates could be expelled in these handling rituals, and if a suspected individual was thus marked by the corpse, guilt was established and that person was in lethal danger. More usually, an occult specialist was consulted, who through trance rituals would commune with the spirit world, and the specialist would then be able to reconstruct the course of occult assault, pointing to various features of the scene as physical traces of an occult sequence. Sometimes a specific culprit could be named, but it was also possible (as with one case described in some detail) for the specialist to declare the death an instance of stranger danger, coming from an unidentified outsider. Knauft describes Gebusi daily interactions as almost always warm, affectionate, and welcoming. Yet the community was darkened by a background fear of witchcraft, and of being accused of witchcraft. In the Gebusi world of the 1970s, the rate of violent death was very high, and the majority of them were associated with witchcraft accusations.

At a coarse level of description, the Australian ethnographic record paints a similar picture. Death by illness (especially) and accident is regarded as likely to be the result of occult attack. Not always: occult forces can act without human intervention if a serious taboo is violated (e.g., trespassing upon sacred burial sites), and deaths of the very old can be regarded as unsuspicious. But the basic pattern is similar. Occult attack is usually the work of outsiders, but it can come from within the social circle (sometimes recruiting outsiders to actually work the sorcery). Keepers of esoteric knowledge of various kinds had high status in Aboriginal Australia. These included occult specialists—“clever men”—who are the main line of defence in diagnosing and repelling occult danger, especially of illness that is the result of malicious magic. In addition to protection from specialists, a kind of occult hygiene was also important, as intimate objects and intimate body products made a person very vulnerable if they fell into the hands of an enemy (as in the principle of contagion).

At a finer grain, the details of occult threat and defence against such threats varied enormously between Australia and elsewhere, and across Australia. J. B. Love (1936/2009), for example, describes a Kimberley diagnostic procedure (in the 1920s), with an occult specialist requiring all relevant adults to place a stone, representing themselves, around a corpse believed to be a victim of sorcery. The stone of the guilty party will become stained with red overnight, placing that individual in serious danger. Love describes at least one subsequent killing, and he takes it for granted that the specialist stained the stone himself. Illnesses that are the result of occult attack can sometimes be cured by counter-magic, as when an occult specialist “detects” a foreign insertion in the victim’s body, placed by occult means, and removes it, showing a twig or some similar small object to suitably impressed onlookers. Unsurprisingly, many early observers (including Love) took this as evidence that the specialists were frauds, and knew themselves to be frauds. But as Elkin (1946) points out in his study of occult specialists, *Aboriginal Men of High Degree*, this can be seen as similar to Western practitioners using methods they knew to be without physical effect, to secure the important benefits of optimism or hope on recovery (a point also made by Hayden (2003) in his more general study of shamanism). That said, many reports suggest serious negative placebo effects: those convinced that their illnesses were the result of irresistible sorcery were much less likely to recover.

A striking feature of these systems is their resilience. Janice Reid’s *Sorcerers and Healing Spirits* (1983) is an ethnography of an Arnhem Land culture, the Yolngu, in the 1970s. As with other examples, the Yolngu believed that death, injury, and other misfortune was the result of malicious sorcery. These sorcerers, *galka*, could influence the action of an array of occult beings who inhabited the landscape to do ill. Galka were typically thought of as outsiders whose personal identity was not known, but individuals in the social world, motivated by spite or resentment could recruit them as allies. In important ways these communities were integrated in the larger Western society, interacting with and using Western technology and even medicine. But as Reid showed, belief in occult risk, and in the role of specialists in defence against occult risk, remained extremely robust.

## The Lessons of Ethnography

We take these case studies to vividly illustrate certain general considerations, ones backed by (much) more extensive surveys of pre-state, small-scale societies in this area (see, e.g., Hutton, 2017; Murdock, 1980; Singh, 2021, Winkelman, 1990).

### These Systems of Belief and Practice Are Widespread

Human cultural variation being what it is, we predict that there are or were examples of cultures free of these beliefs and practices.^7^ But the agential view of misfortune is common: it is found (or is known to have been present) in every continent. The view is not tied to a specific economy: the Australian examples are of mobile foragers; the Gebusi are horticulturalists; the Azande, mixed farmers. There does not seem to be any specific ecological trigger; for example, and perhaps in contrast to increased investment in material technology, the practice does not seem to be a response to increased environmental risk. Some form of the agential view of misfortune seems to be a typical feature of small world communities.^8^

### These Systems of Belief and Practice Reoccur and Are Resilient

We do not think the agential view of misfortune is a preserved accident, evolving once and inherited by cultural descent from a common ancestor. Instead, it is likely that these beliefs and practices have evolved culturally in parallel. If the *sapiens* migration out of East Africa began about 80 kya, that is the minimum time since the Gebusi, Yolgnu, and Azande ancestors were members of the same community, and so the Azande cultural branch is separated from the Gebusi/Yolngu branch by more than 160,000 years of evolution. That is a very long time for cultural legacies to persist. For perspective: many linguists argue that beyond a window of 6,000–10,000 years the rates of language change block our ability to reliably distinguish between lexical similarities among languages that are due to common descent from those that are due to diffusion and/or chance similarity (e.g., Kaufman and Golla 2000; Nichols, 1992; Ringe, 1995; but see Evans, 2020).^9^ Though PNG and Australia were connected by land in glacial times within the continent of Sahul, and were probably colonised by the same peoples sometime before 50 kya, there is remarkably little evidence of cultural or demographic connection between Australia and PNG. The Yolngu and Gebusi may well have been culturally separate for more than 40,000 years. Moreover, the similarities between the three examples are both general and essential to the agential view of misfortune: the availability of occult forces; the ability and willingness of human agents to access and use those forces; the importance of spite, jealousy, and resentment as motivations; the role of occult specialists in diagnosis and defence. These features form an integrated, coherent system. Descent from a shared cultural ancestor is typically shown by shared contingent, inessential features of a practice. Ethnography suggests these are highly variable, though with some medium-scale patterns.^10^ If these considerations against a common origin and a preserved accident are correct, the agential view of misfortune is recurrent and resilient: it is apt to arise and persist in a wide variety of small-scale communities. If Reid’s example is typical, this view is apt to persist even in contact with large social worlds that reject this form of magical thinking. One of us (Sterelny) has firsthand experience of this. A natural history guide on the Sepik River (in the northwest of PNG), rightly proud of his achievement in securing clean water for his village, became visibly distressed when he realised he had lost an old T-shirt. The loss of an intimate garment made him vulnerable to leavings sorcery.

### These Systems of Belief and Practice Impose Serious Costs

Since the Azande live in an effectively policed state, accusations of sorcery do not erupt in violence. Moreover, they have conventions that allow individuals accused of witchcraft to admit guilt, but to claim that they were unaware of being the source of occult trouble, and to take part in rituals to cool their overheated stomach (the presumptive organ of anger). So, for the Azande, the major costs were the added friction to everyday life. Otherwise appropriate plans (trips to the market, hunts, social visits) are abandoned, postponed, or pursued in inefficient ways—for example, choosing long and inconvenient travel routes to avoid close approach to potential sources of occult danger. Likewise, friction costs, and modest material costs, are entailed in consulting oracles and waiting on their responses. Furthermore, though this ideology must be a symptom of limited social trust in Azande circles (since occult threat comes from within), these practices are likely to have exacerbated, or at least entrenched, this lack of trust.

As Reid describes Yolngu lifeways in the 1970s, they paid rather similar costs. Perhaps the most important were opportunity costs. Because *galka* were outsiders, the agential view of misfortune encouraged suspicion of outsiders and thus shrank opportunities for profitable interaction with them. But as with the Azande, and even discounting adverse effects on trust within Yolngu communities, it created friction costs in everyday life too: avoiding being out and about at night, having to guard the disposal of one’s own body products and keeping their location secret, keeping safe anything intimately connected to one’s own body. Occult specialists, too, required support and respect. In general, vigilance is costly when there is no real danger in the offing.

For the Gebusi, the costs were much higher. Accusations of sorcery, often beginning with gossip and a whispering campaign, regularly lead to executions (and these in turn could lead to lethal retaliation). Knauft estimated that about one-third of adult deaths were violent, and that somewhat more than half of these were the result of sorcery accusations. According to his analysis, the root cause of many accusations was reciprocation failure in marriage between extended families. Women who left one such family were not balanced by others coming in, and this created a subterranean resentment despite overt claims that all was well. He suspected that his numbers even underestimated the lethal costs of the agential view of misfortune since other violent deaths were the result of intercommunity feuds, and these sometimes began with sorcery accusations. In Knauft’s data (collected in the 1970s and early 1980s, but with some earlier components), the victims of execution tended (but only tended) to be older vulnerable women, perhaps because they were less likely to attract police attention from reports by aggrieved relatives or result in more direct retaliation.

The Azande, and the Yolngu of Reid’s ethnographic period, were embedded in a larger polity and were effectively policed. The Gebusi were on the fringes of effective policing, and we suggest that the costs the Gebusi paid for their acceptance of the reality of sorcerous malpractice is a better model of the typical cost of these practices (until very recently). Many small pre-state communities combine the view that all/many deaths by accident or illness are actually murders, with a norm of retaliation. An injury inflicted on kith, kin, or clan must be balanced by inflicting an equivalent injury on the source. Jointly, this combination is a recipe for blood feuds. Yet it is typical of, for example, Australian Aboriginal communities. The Yolngu would certainly have endorsed it, and so at an earlier phase of their cultural history, the costs of their belief in the existence of malevolent *galka* would have been much higher.

In sum: the costs of sorcerous belief and the practices surrounding it impose clear social, opportunity, and (to a lesser extent) material costs. In the limit, the costs can be lethal for those on the receiving end. These costs are obviously consequential for individuals who pay them, and that is especially true of conflict costs. Even when they do not lead to violence, fracturing social relationships matters, especially in economies where social support is a critical resource. But they are also important for communities. It is worth reminding ourselves at this point that the loss of a group member can be extremely consequential from a subsistence perspective in such small-scale forager societies (for example, if the deceased was an expert hunter or toolmaker of some kind).

### These Systems of Belief and Practice Are not the Result of Causal Opacity

Some important domains of human action are causally opaque. It is no surprise that most folk medicine is ineffective or worse. With the exception of extreme, immediate effects (most of which will be unfortunate), it is difficult to judge the effectiveness of a medical intervention. Many will recover from illness or injury even if a procedure is inappropriate, and some will die even if an appropriate intervention gave them their best chance. In most cases, we need controlled experiments, large sample sizes, careful recording of all outcomes, and detailed statistical analysis to judge medical effectiveness. Diagnosing cause has required our best technologies from the nineteenth century on. But belief in the power of sorcery and the like cannot be explained by doxastic floundering in the face of causal opacity; far from it. Belief in the power of sorcery extends to death or injury from accidents, and as Evans-Pritchard points out, these agents understand mechanical causal processes. However, they regard these mechanical causal sequences as just the proximate cause of (say) death. If a man dies of snakebite, the locals know that snakes inject venom when they bite, and that this can kill. But why, they ask themselves, was the snake there-then, just as this very man walked by? Occult agency induced it to be there. And, indeed, Spencer (1914) reports a Kakadu occult procedure supposedly producing close encounters between the target of the procedure (targeted by digging up and using some of his/her excrement) and venomous snakes. The irrelevance of causal opacity here is powerfully driven home by the fact that such occult explanations are routinely provided in many cultures even for the most causally *transparent* cases of accident (which, again, are grasped mechanically by the relevant explainers).^11^

Pulling these observations together, then: we think it is clear that belief in the reality of occult murder requires special explanation. These beliefs are false; they are not the result of causal opacity, and they impose costs on those who have those beliefs. Why then are they so common in so many otherwise diverse cultures? This is the critical question. Beliefs are maps “by which we steer” (Ramsey 1929/1990), and, to borrow a felicitous phrase from Godfrey-Smith (1998), accurate representations of the world are a “fuel for success.” All other things being equal, whatever our projects, our prospects are better if the map by which we steer is accurate.^12^ Reciprocally, seriously mistaken representations of our world are fuels for failure, and failure can be costly, even lethal. So we expect selection to have shaped our belief-forming strategies. We do not expect selection to build agents with ideal maps of their world. As Godfrey-Smith points out, it is in general impossible to simultaneously maximise completeness (every detail represented somehow) and reliability (every detail represented correctly). Moreover, accessing, storing, processing, and using information is often costly. We are limited beings: limited in our capacities to use the information we have, and limited in our resources to pursue epistemic action, gathering more. Inevitably, our belief-forming strategies will be heuristics, with some degree of error. But sorcerous beliefs are puzzling even on quite modest views of the power of selection to shape belief-forming strategies. We show in the next section that this puzzle remains even when noting the inevitable limitations on human cognition. Moreover, the typical costs of this system of belief and practice are high, sometimes very high. A heuristic that routinely promoted acceptance of the agential view of misfortune would have to have very impressive compensating benefits, and we argue in the next section that there is no plausible candidate. *Prima facie*, we would have expected this system to have been purged by natural selection, acting on individuals, social groups, or both. It has not, and this requires special explanation.

Importantly: in arguing that the prevalence of the agential view of misfortune requires special explanation, we particularly have in mind the initial *establishment* of these beliefs and practices as the community consensus. Once it is established as consensus, its *persistence* in a community might be explained by the mechanisms of intergenerational cultural learning.^13^ Azande children, as with all others in these communities, learn an enormous amount from the preceding generation. Most of what they learn is useful (as practices) and true (as beliefs). Even so, as Dan Sperber and Hugo Mercier have shown in their work on epistemic vigilance (see, e.g., Sperber et al., 2010; Mercier & Sperber, 2011), we do not play Trust All as social learners (though younger children might). We check the reliability of what we hear. So, even once a consensus on the agential view of misfortune has been established, given its costs, and its evidential impoverishment, it might erode. However, scepticism is resource-intensive (and hence costly), so most of the common beliefs—the accepted stock of local opinion—will be accepted with little or no scepticism. This will typically include fundamental beliefs, such as about the ultimate nature of the cosmos, the sorts of entities and forces it contains, and the group’s position within this order. In many cultures, this set is apt to contain sorcerous belief and the like. Moreover, the tendency to accept sorcerous beliefs may well be amplified by what Henrich (2009) has called *credibility-enhancing displays* on the part of adults. The fact that adults pay significant costs to mitigate or avoid occult danger, often publicly, credibly signals that they genuinely fear such danger. To the extent that these adults are regarded as reliable models, the next generation will conclude the danger is real. It is even possible that once belief in sorcery becomes standard, scepticism might be seen as suspicious, as evidence that the sceptic is a sorcerer, encouraging others to drop their guard.^14^ So, perhaps once this system of thinking and acting becomes entrenched in a community, its persistence may be explained by the central role of intergenerational social learning in human life, together with constraints on individuals to independently test and vet socially displayed information. Once common, it may not pay, on balance, for individuals to sceptically challenge that consensus. However, the mystery of how this system establishes and becomes entrenched remains.

In the next section, we take up the idea that heuristics that are overall adaptive lead to acceptance of the agential view of misfortune.

## Side Effects?

No agent can construct an ideal map of his or her world. We are limited beings, and in forming our view of the world, we must use heuristics with some error rate. But we see no candidate that is likely to be overall adaptive for human agents which would also lead to buying into the agential view of misfortune.

## Occult Agency and Perceptual Trust

One adaptive heuristic is trust in perceptual appearances. This heuristic explains the intuitive appeal of an earth-centred cosmology and the view that the sun moves from east to west. In terrestrial contexts, apparent motion seen by an observer at rest is good evidence of actual motion. If one of us stands on a beach and sees a sea eagle apparently coming from the direction of sunrise, moving overhead and disappearing into the west, they will be right to think that the eagle was in motion, moving from east to west. It is no surprise that naive observers did not distinguish between the apparent motion of terrestrial versus celestial objects. Could perceptual trust explain acceptance of the reality of malicious magic? Occult specialists certainly sometimes manipulate the perceptual attention of onlookers and show physical traces of occult activities, impressing their audiences with their diagnoses (Elkin and Knauft recount examples). But they do so against a belief system that already accepts their expertise as real. Most of the supposed physical residues of occult activity are lame: minor bits of local debris. They are credible only because audiences are primed to believe. It is much harder (though perhaps not impossible) to imagine an individual inventing a deceptive display without the credibility boost of associated, occultist cultural lore and simultaneously using it to convert to belief a large number of onlookers. So, while deceptive displays of this kind—particularly where they are carried out masterfully—may well help solidify or maintain an individual’s reputation as a legit occultist as opposed to an imposter, and while they may help to maintain occultist beliefs in the group more generally, we think it is unlikely that deceptive displays established these beliefs.

## Malign Magic, Causation, Correlation

Another powerful heuristic is using correlation as evidence for causation. Consider, for example, the seventeenth- and eighteenth-century belief that malaria was caused by “bad air”—by spending time in or near miasmic swamps or marshes. Malaria is not caused by breathing humid motionless air, but by mosquitos which breed abundantly in such conditions, so there is a correlation between wetland exposure and catching malaria. Correlation does not establish causation, but it is positive evidence for causal connection (and indeed there is one in the case of malaria, albeit indirect). So, while that diagnosis of malaria’s cause is mistaken, it is not irrational. Singh (2021) claims that one critical factor that leads to belief in malign magic is just such a causal illusion: agents attempt to practice malicious magic, and it appears to work occasionally. Our cognitive system is such that these “successes” are salient and memorable; moreover, there is little penalty for drawing this causal inference since it is not costly. We disagree, even setting aside the issue of cost. First, we have good evidence that almost all small-scale pre-state cultures think that humans cause misfortune by occult means. Evidence that humans *actually attempt* malicious magic comes from a much more restricted set (in Singh’s own sample, about half). Thus, Knauft thinks there are no would-be Gebusi sorcerers. Even in those cultures in which would-be sorcerers practice, the practice is necessarily secret (because it is likely to attract violence) and, we conjecture, expensive or risky for those buying these services. If so, this will limit customer numbers. It follows that few people in these communities will know of specific, targeted attempts when they are made, and since these practices do not work, even fewer have the chance to be impressed by apparent success. That said: it is of course true that in communities in which these practices are prevalent, the laity are impressed by apparent success. In Australian Aboriginal communities, onlookers are impressed when an occult specialist removes a foreign object and declares it to be the source of the occult violence, but as above, this is because they already accept the reality of these forces and the validity of these practices. Their credence is not the result of an admittedly somewhat noisy observed correlation between attempts at occult practice and success. There is no observed correlation between sorcerous attempts and mysterious misfortunes.

On the other hand, the use of “good” magic by shamans or other doctors is on much wider display in small-scale communities. This is not surprising; one would expect individuals to desire credit for positive results “rendered” via such means. And while the correlation between these efforts and individual recuperation is still doubtlessly noisy, the cognitive biases cited by Singh (2021) and placebo effects may indeed work to foster belief in the efficaciousness of the shaman’s practices. This is especially true if and to the extent that shamans are sensitive to natural cues of desired results in deciding whether to take up a particular case. For example, Berndt and Berndt (1988) note that Aboriginal doctors in western Arnhem Land will sometimes “reject a case which they envisage as hopeless,” claiming that they have been “called in too late” (1988:311). Similarly, we can imagine that (e.g.) the rainmakers who are the most apt to impress are the ones who focus their efforts during seasons when rain can be expected anyway, and read natural signs of rain (though it is also true that their bush-wise audience will be alert to the same natural cues). We should not be surprised by this; like stage psychics, shamans very likely possess genuine perceptual and cognitive skills other group members lack—just not the skills and knowledge which they *claim* to have. One possibility, then, is that belief in occult powers has its source in the domain of good or light magic, with those beliefs seeding belief in bad or dark magic. We shall briefly return to this idea below.

## Sorcery and Hyper-Vigilance

Perhaps the most important attempt to explain the agential view of misfortune as a side effect of adaptive reasoning appeals to the idea that different errors have different costs. Some errors have no practical consequences; others are fatal. One important line of thought, initially central in discussions about the evolution of religious belief (see Atran, 2004; Boyer, 2008), focusses on potential cost differentials between false positives and false negatives. This distinction is familiar in medicine. In designing a test for lung cancer, in general it is worse to have detection errors that indicate that a patient’s lungs are tumour-free (a false negative, a false “no tumour”), than ones that indicate the presence of a tumour when none is present (a false positive). Applying this distinction to human evolutionary history, it is proposed that in navigating one’s world, it was more costly to falsely think no one was present than to over-count agents; that is, to think or suspect that someone was watching and acting, when no one was there. Being over-vigilant was less expensive than being under-vigilant since under-vigilance could be immediately fatal. Many animals are extremely wary about the potential presence of predators, and for good reason. Over-caution might cost a meal or delay one; under-caution can cost you your life. Assimilating this idea to the human situation, the suggestion runs that we have evolved to be hyper-vigilant about the presence of agents. Perfection is impossible, and while hyper-vigilance leads to errors, the costs of those errors were lower than the potential price of under-vigilance. This—so the thinking goes—explains the pan-cultural tendency to explain puzzling phenomena such as lightning and thunder as the work of hidden agents. This then extends to misfortune.

The idea that humans are hyperactive agency detectors is often associated with the more general thesis that increases in social complexity have been a crucial driver of human cognition (the “social intelligence hypothesis”). We embrace the latter hypothesis (at least in its generic form). Moreover, we even agree that increases in social complexity very likely selected for certain specialized sociocognitive mechanisms. Nevertheless, explaining the agential view of misfortune in terms of a hyperactive agency detection mechanism remains problematic for at least three reasons. First, and most simply, an innate cognitive bias in favour of agential explanations of misfortune does not in itself explain why *other humans* are so commonly implicated in explanations of misfortune. In many of the cultures in which the agential view of misfortune is prevalent, people take their world to be inhabited by occult beings with their own emotions and intentions, not always benevolent. These beings are often thought to be dangerous, and their haunts are to be avoided. If misfortune must be explained by agential action, it could be explained in terms of malice, mischief, or ill will in the occult world, without human intervention (“as flies to wanton boys are we to the gods; they kill us for their sport”). Such explanations are culturally available in these communities; indeed, it is what we see in cases of pure spirit aggression (Murdock, 1980).

Second, the positive case for adaptive hyper-vigilance is not convincing. Side-effect explanations of belief (and the like) succeed when.

- there is a coherent evolutionary explanation for the existence of the relevant mechanism;
- that mechanism would indeed tend to generate that side effect in its normal operation in standard conditions; and.
- if side effects are costly, no minor tweak in the mechanism would reduce or eliminate them (otherwise we would expect natural selection to fine-tune the mechanism over time).

It is not plausible that belief in occult beings, and especially in occult beings that can be induced to do human bidding, satisfies these conditions. Probably from at least the Middle Pleistocene on, humans were members of the predator guild, and as armed social carnivores, they were an increasingly influential member of that guild. We were predators, not prey. As Louis Leakey said, we were not cat-food (earlier hominins probably were). That changes the relative costs of false positives and false negatives. False negatives are less expensive since cryptic observers are less likely to be a threat. False positives are more costly since missed opportunities matter to predators. For the typical herbivore, food packages are small and common. For the typical large predator, food packages are large and rare. We were large predators, and passing up opportunities to move, explore, and search would have been expensive. From the Mid-Pleistocene on, selection on human psychology was unlikely to favour hyper-vigilance about physical threat (Sterelny, 2018; Planer & Sterelny, 2022).

Singh (2021) argues that hyper-vigilance drives malign magic belief systems. But he argues that for humans, selection for hyper-vigilance was selection for hyper-vigilance about the machinations of other humans. We have doubts about this version, as well. The ethnography of mobile foragers emphasises the physical and social intimacy of their lives, and hence the informational transparency of forager camps. In these contexts, it is very difficult to keep secrets; for example, it is hard to have illicit sexual liaisons because all in camp recognise one another’s tracks (and can estimate when they were made (Nicholas Evans, personal communication). The same is true of secret plotting since dwellings are close together, typically open-sided, and much activity takes place in public spaces. Moreover, while there are plenty of quarrels and complaints in these communities (often over food and sharing; see Weissner, 2014), their relatively egalitarian economic and sexual structure makes it unusual—though by no means impossible—for vital interests to be at stake in disagreements (Australian foragers, with differential age-related sexual access of men to women—see Keen (2004)—may be a partial exception). In short: a combination of serious threat and serious uncertainty about its sources would need to be a standard feature of forager social environments to drive selection for hyper-vigilance. We doubt either condition is routinely satisfied. Moreover, hyper-vigilance is likely to be seriously costly since it frequently results in unwarranted suspicion. This matters, as social capital embedded in a network of mutual obligation between allies is very important to life prospects in these social worlds. The idea that human-focussed hyper-vigilance is or was adaptive in traditional human social worlds thus strikes us as implausible.

We accept that this picture of forager life is no longer uncontroversial (see Singh & Glowacki, 2022). If it is mistaken, ancestral human social lives probably involved more high-stakes issues and more high-stakes social machinations. But these communities would still have been physically intimate, small scale, and by comparison to later social worlds, informationally transparent. Moreover, in a world of more divided loyalties and high social stakes, the costs of false positives, in fracturing relationships and making unnecessary enemies, would have increased. So, even if the standard view exaggerates the egalitarianism of ancestral forager life, the crucial claim made by Singh (2021) of cost asymmetry between excess suspicion and excess trust is unsupported.

Finally, even if agents were adaptively hyper-vigilant, we would not expect hyper-vigilance to be a belief-forming heuristic. In the context of predator-prey interactions, hyper-vigilance is (arguably) an adaptive solution to a perceptual detection problem. A herbivore listening for movement or focussing on a suspicious pattern in the grass faces a detection problem—a perceptual puzzle that must be solved quickly and acted on immediately, in one of a few ways. Especially if Singh is right in thinking that the background threat is social machination, not physical ambush, human agents face a different detection problem. Against threats of social machination, we might predict enhanced alertness to cues of danger (for example, changes in the warmth of social interaction); greater investment in tracking third-party social interactions; greater investment in epistemic action (gossiping with others, eavesdropping); investment in affiliative relations (making sure you share generously with friends, being careful to avoid pointless complaints), and so on. If agents really do live in social worlds in which enemies plot behind their back, belief in the reality of occult agents that can be recruited to do you harm is unlikely to help. Indeed, it may even hurt: e.g., leaving camp to carefully conceal where you urinate really does allow others to gossip behind your back. Resources are finite, so guarding against nonexistent threats leaves an agent more open to real ones. We do not think this is a plausible picture of ancestral social environments, but if it were, the adaptive challenge would not be solved by a cognitive strategy leading to enduring, revision-resistant belief in occult agents.

Our conclusion restates that of “The Lessons of Ethnography”: the action of selection on human cognition is constrained in many ways, but one would expect selection to purge human minds of the agential view of misfortune, given the costs of that view. This is the fundamental puzzle of belief in malign magic. In the next section, we explore a solution that depends not on constraints on rational belief formation but on communication, and the power of one agent to shape the views of others.

## Malign Magic: Who Pays? Who Benefits?

Belief guides behaviour, with variation in the choice of action sensitive to variation in belief. Decisions about what plants to collect and process will be sensitive to beliefs about the location of different plants, the costs of their collection and processing, and the value of products that can be made from them. The metaphor of the map by which we steer captures this sensitivity of plan to belief, and the relationship between plans succeeding and the accuracy of those beliefs. However, belief can affect life prospects in other ways. Beliefs (when expressed or made manifest in behaviour) can serve as social signals, expressing character, commitment, and identity, while also motivating action congruent with character, commitment, and identity (Sterelny, 2015). If someone expresses the belief that Trump was the true victor of the 2020 US election and if he in other respects acts in conformity to that belief (he wears his MAGA hat with pride; he dons his politically incorrect T-shirt picturing Biden behind prison bars), he sends a social signal to others. In the right context, that signal can be rewarding independently of facts about the conduct of that election. There are contexts in which those social rewards dwarf any costs from actions that misfire because they are based on a false view of the world. This is especially true for the many beliefs we have which we do not much use in steering particular courses of action, actions that succeed or fail according to whether we have the correct view of our world. The aforementioned MAGA believer is unlikely to execute a plan that will succeed if Trump was the true winner, but fail otherwise. Expressed beliefs can also shape others’ actions (e.g., others’ willingness to select one as a partner for a cooperative venture), and those actions can benefit (or harm) the agent independently of the truth of the expressed beliefs. These social effects of belief make it possible for there to be special classes of belief that are both false and adaptive, and a substantial fraction of those working on the evolution of religion hold that religious belief falls into this class (see, e.g., Henrich, 2009; Sosis, 2006; Sosis & Alcorta, 2003; Wilson et al., 2014). The details of the different hypotheses vary, but the common theme is that religious belief and its expression enhances social trust by signalling and reinforcing common social identity, and that it enhances norm compliance both via intensifying subjective commitment to the correctness of the norms and through enhanced fear of punishment for violation (even though that fear is founded on an inaccurate appreciation of danger).

The beliefs associated with the agential view of misfortune obviously overlap with those involved in religious practice and belief. This overlap includes belief in supernatural beings and forces; belief that those beings are often aware of, and not indifferent to, human activity; and the belief that these beings and forces can be influenced by human action. Given that overlap, it is unsurprising that adaptive models of religion have been extended to belief in malign magic. The simplest version of this idea, that beliefs and practices around malign magic have benefits similar to those around religion, strikes us as implausible (though it has been defended; see Singh, 2021, for citations and a suitably sceptical discussion). One critical issue is that occult threat is often understood as originating from within one’s social group, in which case it works to erode rather than enhance within-group social cohesion. We saw this with both the Azande and Gebusi. Such cases are quite frequent (for others, see Bubandt, 2006; Geertz, 1960; Heckenberger, 2004; Herriman, 2007, 2012; Lovric, 1986; Peletz, 1993; Rowlands & Warnier, 1988; Stasch, 2001; Wessing, 1996; Wikan, 1987).^15^ Moreover, occult attacks are believed to misfire; one may wind up the victim of an occult attack that was intended elsewhere. As Reid reports: “Cases of mistaken identity and botched or misdirected sorcery abound in firsthand [i.e., victims’] accounts” (1983:113). Finally, and just as importantly, norm compliance is not seen as a sufficient defence against malign magic. Rather, occult hygiene (e.g., burying bodily wastes), and the aid of occult specialists in precaution, defence, and amelioration, must be recruited toward this end.

That said, this system of belief and practice clearly benefits occult specialists. One Gebusi example discussed at some length by Knauft is particularly telling. He describes a death in the forest, one he takes to be accidental. The man in question was known to have an especially tempestuous relationship with his wife, so his death alone in the forest would normally make her the prime suspect. After suitable rituals and trance, the occult specialist investigated the scene of death, and cleared her of suspicion, declaring that the attack had come from outside, finding various occult traces of the attack and attackers, following a supposed trail until it faded out. Subsequently, the ex-wife in question provided the specialist with sexual services. This is not an isolated case. Love is admittedly a hostile witness, but even so, he reports the Kimberley community as having a mix of respect and fear of occult specialists. Elkin (1946) is more sympathetic, but even the title of his *Aboriginal Men of High Degree* shows the social prestige that accrues to occult specialisation. Prestige is valuable: Henrich and Gil-White (2001) show that prestige in these small-scale societies often brings material benefits. Writing five decades after Elkin, Keen (1997) has shown that in many Australian Aboriginal communities, control of esoteric knowledge confers high status, and high status yields sexual access. In a very different geographical context (the most southern region of South America), Lucas Bridges (1948) documents control of women and uninitiated men by initiated Fuegians exercised through fear of occult beings, and the belief that only initiated men can provide protection. In short: the belief in occult practices is exploitable, exploited, and brings serious benefits to those exploiting.

This is a piece of the puzzle, but it is well short of an explanation. If this move is available in the competitive world of social interaction, some agents will choose it. But how does it come to be available, and why do we see so little sales resistance from the exploited majority? (Indeed, in some cases, communities even apply strong social pressure on specific individuals to take up a specialist occult role.) The ethnography of malign magic suggests an arms race with only one side racing. One partial, possible explanation is that the exploitative strategy is parasitic on the prior evolution of religious practices and their associated beliefs. These establish a background picture of the world with occult beings sometimes attending to, and being influenced by, humans. Moreover, these occult beings are by no means always benevolent. A conceptual framework emerges in which physical and biological phenomena are often the result of intervention by these occult beings. On such a conceptual framework, there is less of a gap, though still a considerable one, between that initial conception of the world and one in which (a) most human misfortunes are the result of actions by occult beings (or other esoteric forces); (b) these actions are not the autonomous choice of such beings, but instead have been induced by ill-willed human agents; and (c) to some extent, occult specialists can warn of and/or ward off these dangers.

We think it is probable that the agential view of misfortune is parasitic on the prior evolution of shamanistic belief systems, and we do think it is plausible that there is some cognitive bias in favour of agential causal explanations of puzzling phenomena, perhaps linked to what seems to be an extreme unwillingness to recognise one’s total ignorance about the causes of these phenomena.^16^ We have read ethnographies in vain searching for reports of communities that respond, when asked about the causes of (e.g.) lightning, with “We have no idea. We were hoping you might know.” But this bias is part of what needs explanation. In discussion, it has regularly been put to us that there is profound psychological discomfort in feeling that one is just the victim of the random play of events.^17^ Believing that “shit happens” is a luxury good, accepted only in rich societies where shit does not happen very often. Acceptance of the reality of malign magic is part of a system of belief that gives agents an illusion of control, alleviating this discomfort. Perhaps so, but even if we accept this psychological diagnosis, it bears emphasis that this is not an adequate explanation given the serious material costs of these beliefs—costs that often include blood feuds. We *do* live in an indifferent universe. The illusion that we do not imposes serious costs. The discomfort of acknowledging the world’s indifference is part of the puzzle. Just as, all else being equal, we expect selection against belief-forming strategies that result in costly false beliefs, we also expect selection against credulity-inducing emotions that lead to false, costly, exploited beliefs. What makes this illusion valuable or, alternatively, what makes it such a stable feature of the human psyche over so many different social environments? Without answers to these questions, this psychological vulnerability is part of the puzzle of the lack of sales resistance.

## Sales Resistance

Our sketch of an explanation of the agential view of misfortune would predict sales resistance, yet there seems to be very little. We suggest three possible diagnoses of this surprising fact. The most mundane is that we are simply mistaken about the overall distribution of costs and benefits. The agential view of misfortune clearly advantages occult specialists offering diagnostic and protective services. But perhaps for most other agents, most of the time, the cost of this system of belief and practice is fairly low. So while selection acts powerfully on (would-be) occult specialists to sell their ideas about the danger of occult harm vividly and compellingly, it acts less powerfully on most of the audience to be sceptical. This is akin to some combination of Dawkins and Krebs’s (1979) life-dinner principle (whereby prey species may experience stronger selection than their predators) and Dawkins’s (1982) rare enemy effect (whereby a rare enemy exploits the prey’s response to its primary predator). Nothing is free, so selection does not favour heavy investment against small and/or very occasional dangers. Moreover, Sperber and Mercier’s work on epistemic vigilance suggests that effective sales resistance might be quite expensive (see, e.g., Sperber et al., 2010; Mercier & Sperber, 2017).

One idea, common in anthropological circles, is that those who are not occult specialists can sometimes still exploit it by using accusations of malign magic for their own strategic ends (see, e.g., Peacey et al., 2022, and Micheletti et al., 2023, for a recent discussion) and, in so doing, lower the costs of this framework. That may well be. Once this framework exists, agents will seek to exploit it. But these interactions seem zero-sum: if some non-specialists gain, they do so at the expense of others. An alternative version of this idea has been developed by Boyer (2022), who suggests that these beliefs reduce the community’s responsibility to help a sick or injured person. The idea is that these victims of occult attack have somehow brought these misfortunes on themselves, so only their most intimate associates have a duty of care. Others have a publicly sanctioned excuse to avoid helping, or to give less help. This seems to us to be a more plausible explanation of other occult situations—for example, those involving taboo violations or ancestor anger, whether the victim really can be seen to have brought misfortune on themselves. For victims of sorcery are not routinely blameworthy. Moreover, on this view it is somewhat surprising that agential explanations are so commonly provided in cases of *death*, where no sick/injured individual is consuming resources. These ideas are clearly worth exploring further, though we think the ethnography of the Gebusi speaks against these possibilities since it suggests that the costs to audiences are neither small nor rare. But it is one possibility.

A second possibility is that we have overestimated the power of selection to adaptively shape belief-forming strategies. Perhaps it is much more difficult to purge human minds of a bias in favour of agential explanations, or a bias in favour of endorsing some explanation of puzzling phenomena, rather than confess complete ignorance. These biases (if they conduce to belief in malign magic) lead to false and costly beliefs. But perhaps human minds without these biases are not just a tweak or two away from actual ones. They are not adjacent possibilities, but rather would require a radical re-engineering of the human mind. We are sceptical for a few reasons. (i) Agents in large-scale societies have these biases to much lesser extents. Moreover, this is true despite the fact that, as noted at the outset of this article, many of these same agents are nevertheless prone to less agential but still superstitious thinking (e.g., they believe the number 13 is unlucky; they are reluctant to walk under ladders). This is important because it shows the agential view of misfortune can be and is decoupled from other forms of superstition. Perhaps it is hard to purge all superstitious thinking from our minds, but apparently not superstitious thinking of the agential variety. (ii) Human belief-forming strategies depend significantly on external scaffolds and interactions with other agents (i.e., belief forming is often collective), and this expands our possibility space (see, e.g., Clark, 2006, 2008). For example, it has made science possible. (iii) We have good evidence that in general human cognitive mechanisms have a good deal of adaptive plasticity. Importantly, this includes cognitive mechanisms that underlie thinking about the (potential) role of agents in causing misfortunes (theory of mind, causal reasoning).^18^ (iv) It is a theme of many of the anthropological works discussed here that the people do not *always* appeal to magical or mystical factors to explain life’s happenings, including various misfortunes. For example, while an Azande man may well posit witchcraft as the ultimate explanation for why he hurt his foot while out walking, he is unlikely to do so unless the injury is severe or he suffers a serious infection. More generally, people are happy to accept a non-mystical explanation for many events. So, it is not as though a “shit happens” attitude is cognitively off-limits to them. But all this having been said, we do accept that a constraints-based explanation of some kind for the agential view of misfortune remains a live option.

Finally, the agential view of misfortune may be the result of a multigeneration bait-and-switch. In important cases, intergenerational flows of skill, belief, and opinion have an oblique structure, with a few agents in generation *N* playing a focussed role in the flow to agents in generation *N* + 1. This is often adaptive for all concerned: those with real expertise are the main models for the next generation, and those experts benefit through esteem and respect. But this structure makes bait-and-switch possible. In the bait phase, the intergenerational organisation of information flow establishes in a context in which it reliably transits opinions and practices that are beneficial for the novices: real expertise is made available in return for respect. These models have both trust and respect. But if the interests of those playing this pivotal role in the flow of information differ from those in the rest of the community, biases in the flow of opinion favourable to those playing a privileged role are apt to creep in. This is the switch phase. For example, in traditional Australian Aboriginal cultures, a class of “elders”—those initiated into the full esoteric knowledge of the culture—play the central role in transmitting the norms and values of the culture, and access to this information is essential for a good life in these communities. It is essential especially if those norms and myths encode critical ecological information in salient, memorable ways, as has been suggested (see Kelly, 2015, but for scepticism, see Hiscock, 2020). Given their privileged role in the flow of knowledge, it is little surprise that these norms and values have come to include rights of special sexual access for those elders. Bait-and-switch requires no conscious conspiracy—just the incremental operation of Mercier and Sperber’s (2017) “my-side” bias, our mundane tendency to endorse opinions that favour us.

In previous work, one of us (Sterelny) has argued that this bait-and-switch mechanism played a central role in the decline of egalitarian norms in early sedentary communities, and in changes in the content and costs of religion in the Neolithic. Perhaps something similar explains the widespread acceptance of the agential view of misfortune: it is a transformed and biased version of the shamanism package. Why think this? We leave a full answer to this question for another day and instead limit ourselves to the following preliminary remarks for now. Hayden (2003) has argued that some form of shamanistic practice was common to many small pre-state cultures. These practices have repeated themes: entering trances, inducing altered states of consciousness, and apparent contact with and often help from a spirit world. A crucial difference, in our view, between the “good” or “light” magic of shamans and the “evil” or “dark” magic of sorcerers is that only the former is typically on wide display in human communities; shamans *want* others to behold their efforts to heal. What this means is that a critical prerequisite for the formation of causal illusions via adaptive reasoning heuristics is satisfied. Moreover, Hayden argues that trust in shamans and belief in the reality of their contact with the spirit world was often adaptive, playing a positive role in healing through powerful placebo effects. To us, these features of shamanism are important pieces (though still only pieces) of an explanation for how the practice and beliefs surrounding shamanism could take root in a cultural group. The question of origins is, arguably, less puzzling than in the case of widespread belief in malign magic.

In an important recent article, André et al. (2023) call for more theoretical attention to be paid to the role of *producers’* interests—not just those of consumers—in cultural evolution (see also Boyer, 2021). That paper provides a general framework for thinking about the interaction of producer and consumer interests in the generation of novel cultural forms. They distinguish between cases of *altruistic production* (costly for producers, beneficial for consumers), *selfish production* (beneficial for producers, costly for consumers), and *cooperative production* (beneficial for both, either immediately or in the longer run). They cite shamans, diviners, and priests as plausibly examples of selfish producers. Our “bait-and-switch” cases correspond to modes of cultural transmission that start out as cooperative and over time become selfish. While they regard shamanism as an example of selfish production, to the extent that it offers some benefits to consumers, as Hayden suggests, it may instead be a case of cooperative production. That said, André et al. (2023) are certainly right that the interests of the two parties—producers and consumers—are distinct in the case of shamanism; shamans no doubt benefit a good deal more from others’ beliefs in the authenticity of their powers than do consumers. To this we would add that these interests diverge more—much more, in fact—in cases of sorcery.

There is clearly some overlap between the machinery of shamanism and the work of many occult specialists who claim to deal with malign magic. Indeed, Reid (1983) reports that, while the Yolungu of Arnhem Land neatly distinguish between the two types of specialists in the abstract, that distinction is murkier in the discussion of real-life cases, and she relays one case of a well-known healer (*marrggiti*) who “both claims and is claimed to heal and perform sorcery” (1983:86). Thus, it is not hard to imagine pathways from a preexisting acceptance of shamanism in a community to belief in the possibility of malign magic, and in the efficacy of occultist efforts claimed to protect one from such magic. The end result would in effect be the establishment of an occultist “protection racket.” Moreover, in addition to explaining certain general commonalities in the contents of beliefs about shamanism and malign magic—for example, that both shamans and sorcerers are capable of leaving behind their physical bodies and making journeys in a spirit realm—this current proposal promises to illuminate the distinctively *human* dimension of explanations based on the agential view of misfortune. A belief in shamanism primes the idea that at least some *human agents* are capable of intervening on the world via mystical means (whether assisted in these efforts by occult beings/forces or not). In other words, it makes belief in *human* mystical agency plausible. If so, then there is no need here to invoke a hyperactive agency detection mechanism in order to explain this feature of the agential view of misfortune.

At this stage, we do not commit ourselves to any of these suggestions (though we do favour the last): they are all schematic. For this reason, we take ourselves to have presented only a partial solution to the challenge posed by the agential view of misfortune.

## Final Summary

In summary then, we have argued that (i) the agential view of misfortune is both widespread and expensive; sometimes very expensive; (ii) the beliefs involved are not readily explained as consequences of normally reliable belief-forming strategies, nor as side effects of other adaptive mechanisms; so (iii) their persistence is puzzling, even on quite modest assumptions about the power of selection to purge maladaptive belief-forming practices. The agential view of misfortune thus demands special evolutionary explanation. This is our most important conclusion. The rest of the article has explored a potential, though partial, solution: the agential view of misfortune does not have adaptive consequences for those who have those beliefs, but it does pay for those who induce them. The agential view of misfortune is the result of a manipulation/sales resistance arms race, but one in which there has been little resistance to manipulation. We have considered three suggestions to explain this lack of buyer scepticism (finding one more promising than the others), but they are all preliminary possibilities, and so our solution remains partial.

## Data Availability

Data sharing not applicable to this article as no datasets were generated or analysed during the current study.

## Footnotes

1 See, e.g., Forsyth & Eves, 2015; Geschiere, 1997, 1998, 2001; Herriman, 2006; Schwoerer, 2017; Taylor, 2015; Whitaker, 2017, among many others.

2 We thank a referee for emphasizing this to us.

3 See Hong (2022, 2024) for recent discussions of these alternative types of supernatural explanation.

4 Likely, in part, because these misfortunes may be much more consequential in such societies. See Boyer, 2021, and especially 2022.

5 Boyer (2021) likewise draws attention to the need for special explanation here. See also André et al. (2023).

6 We do, however, set aside group-selectionist logic since group selection is not a plausible mechanism explaining the agential view of misfortune given the latter’s deleterious effects on within-group social relations.

7 In Singh’s (2021) ethnographic survey, only two such societies were identified. An earlier survey by Murdock (1980), however, reported several additional instances of traditional societies in which a belief in “magical causation” of harms brought about by human agents was apparently absent.

8 Of course, this is not to say that beliefs of this kind are strictly limited to such social settings; see, e.g., Wessing (1996) and Bubandt (2006) for examples of belief in sorcery in a much more modern setting.

9 Greenhill et al. (2010) for a general discussion of this idea and related matters.

10 For example, it is commonly thought that those who practice malign magic also break even more fundamental norms—they practice cannibalism, they have sex with animals or devils, and the like.

11 We thank a referee for emphasizing this point to us.

12 See McKay and Dennett (2009) for a discussion of some candidate cases of adaptive misbelief. The phenomenon of self-deception is also relevant here, though self-deception is probably only a partial case of an “inaccurate map.” As Trivers and colleagues analyse the phenomenon, for example, it involves the simultaneous presence of accurate (or true) and inaccurate (or false) representations in different parts of the mind (Butterworth et al., 2022; Trivers, 2011; Von Hippel & Trivers, 2011). Also see our discussion of religious belief, below.

13 For more on how and when origins and persistence/stability can pose distinct explanatory challenges, see Sterelny (2018). An intuitive way to see that the two phenomena can indeed pose distinct challenges is to simply note that the fitness profile of a trait (e.g., religiosity) can differ depending on how common the trait already is in the population (especially among the adult class). For example, those with the trait might impose (social) costs on those without it. The costs of avoidance are then tied to the frequency of the trait in the population. More generally: it is a familiar point from the literature on formal models of the evolution of social behaviour that there are often multiple equilibrium distributions of behavioural strategies in a population. In such cases, a separate explanation is needed to explain why one of these equilibrium distributions has been reached rather than others.

14 The fifteenth-century work *Malleus Maleficarum* explicitly treated scepticism regarding witches and witchcraft as suspicious, labelling those who were sceptics as heretics. Apparently, it was not uncommon for those voicing scepticism to themselves be deemed witches.

15 It may well be that beliefs in others’ malign magic had their original home in the out-group context and only spread to the in-group context later. We remain neutral on this possibility and doubt whether the surviving record gives enough empirical traction to assess its probability.

16 See Horton (1967) for a discussion of the explanation-seeking drives of many traditional African cultures, and how they compare with those underlying Western scientific inquiry. One unique aspect of explanations of misfortune in small-scale societies (as compared with the sorts of explanations provided by Western science) is the focus on *particulars*—why was *this* man bitten by a snake on *this* day (etc.). See Boyer (2021) for a discussion of the particular nature of such explanations.

17 Though with important individual variation: social psychologists have long noted significant differences in the extent to which individuals desire control over situations (see Burger, 2013, for an extended discussion).

18 See Heyes (2018) for a nice and relatively recent overview of such evidence. Heyes draws the strong conclusion that this evidence suggests human theory of mind and causal reasoning mechanisms are themselves culturally constructed mechanisms—“cognitive gadgets” in her terminology. One might recognise a central role for cultural learning in shaping these mechanisms without endorsing a full-blown cultural gadget thesis.

## References

[CR1] André JB, Baumard N, Boyer P (2023). Cultural evolution from the producers’ standpoint. Evolutionary Human Sciences.

[CR2] Atran, S. (2004). *In gods we trust: The evolutionary landscape of religion*. Oxford University Press.

[CR3] Berndt, R. M., & Berndt, C. H. (1988). *The world of the first Australians. Aboriginal traditional life: Past and present*. Aboriginal Studies Press.

[CR4] Boyer, P. (2008). *Religion explained*. Random House.

[CR5] Boyer P (2021). Deriving features of religions in the wild: How communication and threat-detection may predict spirits, gods, witches, and shamans. Human Nature.

[CR6] Boyer P (2022). Why we blame victims, accuse witches, invent taboos, and invoke spirits: A model of strategic responses to misfortune. Journal of the Royal Anthropological Institute.

[CR7] Bridges, E. L. (1948). *Uttermost part of the earth*. Hodder & Stoughton.

[CR8] Bubandt N (2006). Sorcery, corruption, and the dangers of democracy in Indonesia. Journal of the Royal Anthropological Institute.

[CR9] Burger, J. M. (2013). *Desire for control: Personality, social and clinical perspectives*. Springer Science & Business Media.

[CR10] Butterworth J, Trivers R, von Hippel W (2022). The better to fool you with: Deception and self-deception. Current Opinion in Psychology.

[CR11] Clark A (2006). Language, embodiment, and the cognitive niche. Trends in Cognitive Sciences.

[CR12] Clark, A. (2008). *Supersizing the mind: Embodiment, action, and cognitive extension*. Oxford University Press.

[CR13] Dawkins, R. (1982). *The extended phenotype: The long reach of the gene*. Oxford University Press.

[CR14] Dawkins R, Krebs JR (1979). Arms races between and within species. Proceedings of the Royal Society of London B: Biological Sciences.

[CR15] Elkin, A. P. (1946). *Aboriginal men of high degree*. Reprinted in 1977 by University of Queensland Press.

[CR16] Evans, N. (2020). Time, diversification, and dispersal on the Australian continent: Three enigmas of linguistic prehistory. In M. Crevels, & P. Muysken (Eds.), *Language dispersal, diversification, and contact* (pp. 116–141). Oxford University Press.

[CR17] Evans-Pritchard, E. E. (1937). *Witchcraft, oracles, and magic among the azande*. Clarendon.

[CR18] Forsyth, M., & Eves, R. (2015). The problems and victims of sorcery and witchcraft practices and beliefs in Melanesia: An introduction. In M. Forsyth, & R. Eves (Eds.), *Talking it through: Responses to sorcery and witchcraft beliefs and practices in Melanesia* (pp. 1–19). ANU Press.

[CR19] Frazer, J. G. (1990). [1890] Sympathetic magic. In *The Golden Bough*. Palgrave Macmillan. 10.1007/978-1-349-00400-3_3.

[CR20] Geertz, C. (1960). *The religion of Java*. University of Chicago Press.

[CR21] Geschiere, P. (1997). *The modernity of witchcraft: Politics and the occult in postcolonial Africa*. University of Virginia Press.

[CR22] Geschiere P (1998). Globalization and the power of indeterminate meaning: Witchcraft and spirit cults in Africa and East Asia. Development and Change.

[CR23] Geschiere, P. (2001). Witchcraft and new forms of wealth: Regional variations in South and West Cameroon. In P. Clough, & J. P. Mitchell (Eds.), *Powers of good and evil: Social transformations and popular belief* (pp. 43–76). Berghahn Books.

[CR24] Godfrey-Smith, P. (1998). *Complexity and the function of mind in nature*. Cambridge University Press.

[CR25] Greenhill SJ, Atkinson QD, Meade A, Gray RD (2010). The shape and tempo of language evolution. Proceedings of the Royal Society B: Biological Sciences.

[CR26] Hayden, B. (2003). *Shamans, sorcerers, and saints: A prehistory of religion*. Smithsonian Books.

[CR27] Heckenberger, M. (2004). The wars within: Xinguano witchcraft and balance of power. In *In darkness and secrecy: The anthropology of assault sorcery and witchcraft in Amazonia*, edited by N. Whitehead and R. Wright, 179–201. Durham: Duke University Press.

[CR28] Henrich J (2009). The evolution of costly displays, cooperation, and religion: Credibility enhancing displays and their implications for cultural evolution. Evolution and Human Behavior.

[CR29] Henrich J, Gil-White FJ (2001). The evolution of prestige: Freely conferred deference as a mechanism for enhancing the benefits of cultural transmission. Evolution and Human Behavior.

[CR30] Herriman N (2006). Fear and uncertainty: Local perceptions of the sorcerer and the state in an Indonesian witch-hunt. Asian Journal of Social Science.

[CR31] Herriman, N. (2007). *A din of whispers: Community, state control, and violence in Indonesia*. PhD thesis, University of Western Australia.

[CR32] Herriman N (2012). Sorcery, theft and affinity: The estrangement of intimacy in Eastern Java. The Asia Pacific Journal of Anthropology.

[CR33] Heyes, C. (2018). *Cognitive gadgets: The cultural evolution of thinking*. Harvard University Press.

[CR34] Hiscock P (2020). Mysticism and reality in Aboriginal myth: Evolution and dynamism in Australian Aboriginal religion. Religion Brain & Behavior.

[CR35] Hong Z (2022). Ghosts, divination, and magic among the Nuosu: An ethnographic examination from cognitive and cultural evolutionary perspectives. Human Nature.

[CR36] Hong Z (2024). The cognitive origin and cultural evolution of taboos in human societies. Journal of the Royal Anthropological Institute.

[CR37] Horton R (1967). African traditional thought and Western science. Africa.

[CR38] Hutton R (2004). Anthropological and historical approaches to witchcraft: Potential for a new collaboration?. The Historical Journal.

[CR39] Hutton, R. (2017). *The witch: A history of fear, from ancient times to the present*. Yale University Press.

[CR40] Kaufman, T., and Golla V (2000). Language groupings in the New World: Their reliability and usability in cross-disciplinary studies. In C. Renfrew (Ed.), *America past, America present: Genes and languages in the Americas and beyond* (pp. 47–57). McDonald Institute for Archaeological Research.

[CR41] Keen, I. (1997). *Knowledge and secrecy in an Aboriginal religion*. Oxford University Press.

[CR42] Keen, I. (2004). *Aboriginal economy and society: Australia at the threshold of colonisation*. Oxford University Press.

[CR43] Kelly, L. (2015). *Knowledge and power in prehistoric societies*. Cambridge University Press.

[CR44] Knauft, B. (2015). *The Gebusi: Lives transformed in a rainforest world*. Waveland.

[CR45] Love, J. R. B. ([1936] 2009). *Kimberly people: Stone-age bushmen of to-day*. David Welch.

[CR46] Lovric BJA (1986). The art of healing and the craft of witches in a ‘hot earth’ village. Review of Indonesian and Malayan Affairs.

[CR47] McKay RT, Dennett DC (2009). The evolution of misbelief. Behavioral and Brain Sciences.

[CR48] Mercier H, Sperber D (2011). Why do humans reason? Arguments for an argumentative theory. Behavioral and Brain Sciences.

[CR49] Mercier, H., & Sperber, D. (2017). *The enigma of reason*. Harvard University Press.

[CR50] Micheletti, A. J., Brandl, E., Zhang, H., Peacey, S., & Mace, R. (2023). Cultural evolution research needs to include human behavioural ecology. In Agathe du Crest, Martina Valković, André Ariew, Hugh Desmond, Philippe Huneman, Thomas A. C. Reydon (Eds.), *Evolutionary thinking across disciplines: Problems and perspectives in generalized Darwinism* (pp. 501–528). Cham: Springer International.

[CR51] Murdock, G. P. (1980). *Theories of illness: A world survey*. University of Pittsburgh Press.

[CR52] Nichols, J. (1992). *Linguistic diversity in space and time*. University of Chicago Press.

[CR54] Peacey S, Campbell OL, Mace R (2022). Same-sex competition and sexual conflict expressed through witchcraft accusations. Scientific Reports.

[CR53] Peletz, M. G. (1993). Knowledge, power, and personal misfortune in a Malay context. In C. W. Watson and R. Ellen (Eds.), *Understanding witchcraft and sorcery in Southeast Asia* (pp. 149–177). Honolulu: University of Hawaii Press.

[CR55] Planer RJ, Sterelny K (2022). The costs of magical thinking and hypervigilance: A comment on Singh 2021. Current Anthropology.

[CR56] Ramsey, F. P. (1929/1990). General propositions and causality. In D. H. Mellor (Ed.), *F. P. Ramsey: Philosophical papers* (pp. 145–163). Cambridge University Press.

[CR57] Read KE (1954). Cultures of the central highlands, New Guinea. Southwestern Journal of Anthropology.

[CR58] Reid, J. (1983). *Sorcerers and healing spirits: Continuity and change in an Aboriginal medical system*. Australian National University Press.

[CR59] Ringe D (1995). “Nostratic” and the factor of chance. Diachronica.

[CR60] Rowlands M, Warnier JP (1988). Sorcery, power and the modern state in Cameroon. Man.

[CR61] Schwoerer T (2017). Sorcery and warfare in the eastern highlands of Papua New Guinea. Oceania.

[CR62] Singh M (2021). Magic, explanations, and evil: The origins and design of witches and sorcerers. Current Anthropology.

[CR63] Singh M, Glowacki L (2022). Human social organization during the Late Pleistocene: Beyond the nomadic-egalitarian model. Evolution and Human Behavior.

[CR64] Sosis R (2006). Religious behaviors, badges, and bans: Signaling theory and the evolution of religion. Where God and Science Meet: How Brain and Evolutionary Studies Alter Our Understanding of Religion.

[CR65] Sosis R, Alcorta C (2003). Signaling, solidarity, and the sacred: The evolution of religious behavior. Evolutionary Anthropology.

[CR66] Spencer, B. W. (1914). *The native tribes of the Northern Territory of Australia*. Macmillan.

[CR67] Sperber D, Clément F, Heintz C, Mascaro O, Mercier H, Origgi G, Wilson D (2010). Epistemic vigilance. Mind & Language.

[CR68] Stasch R (2001). Giving up homicide: Korowai experience of witches and police (West Papua). Oceania.

[CR69] Sterelny K (2015). Content, control and display: The natural origins of content. Philosophia.

[CR70] Sterelny K (2018). Religion re-explained. Religion Brain & Behavior.

[CR71] Taylor JP (2015). Sorcery and the moral economy of agency: An ethnographic account. Oceania.

[CR72] Trivers, R. (2011). *Deceit and self-deception: Fooling yourself the better to fool others*. Penguin UK.

[CR73] Von Hippel W, Trivers R (2011). The evolution and psychology of self-deception. Behavioral and Brain Sciences.

[CR74] Vyse, S. (2019). *Superstition*. Oxford University Press.

[CR77] Wiessner P (2014). Embers of society: Firelight talk among the Ju/’hoansi bushmen. Proceedings of the National Academy of Sciences.

[CR75] Wessing R (1996). Rumours of sorcery at an Indonesian university. Journal of Southeast Asian Studies.

[CR76] Whitaker JA (2017). Guns and sorcery: Raiding, trading, and *kanaima* among the Makushi. Tipití: Journal of the Society for the Anthropology of Lowland South America.

[CR78] Wikan U (1987). Public grace and private fears: Gaiety, offense, and sorcery in northern Bali. Ethos.

[CR79] Wilson MS, Bulbulia J, Sibley CG (2014). Differences and similarities in religious and paranormal beliefs: A typology of distinct faith signatures. Religion, Brain &amp; Behavior.

[CR80] Winkelman M (1990). Shaman and other magico-religious healers: A cross- cultural study of their origins, nature and social transformation. Ethos.

